# Environmental regulation effect on the different technology innovation-based the empirical analysis

**DOI:** 10.1371/journal.pone.0296008

**Published:** 2024-01-05

**Authors:** Lihua Ma, Shiya Ma, Qisheng Tang, Mingmei Sun, Huizhe Yan, Xiuling Yuan, Wei Tian, Yufei Chen

**Affiliations:** 1 School of Management Engineering and Business, Hebei University of Engineering, Handan, 056009, China; 2 School of Finance, Shanghai University of Finance and Economics, Shanghai, 200433, China; 3 School of Economics, Liaodong University, Dandong, 118001, China; 4 Uibe Business School, University of International Business and Economics, Beijing, 100105, China; IBS Hyderabad: ICFAI Business School, INDIA

## Abstract

This article explores the impact mechanism of different types of environmental regulations on corporate green technology innovation (GTI). The research focuses on analyzing three types of environmental regulations: command based environmental regulation (ER1), market-oriented environmental regulation (ER2), and voluntary environmental regulation (ER3), and how they affect corporate GTI. This study selected enterprise GTI as the dependent variable and measured it by the number of applications for green invention patents and green utility model patents. The independent variables are the three types of environmental regulations mentioned above. According to data from Chinese A-share listed companies. Using benchmark regression models to analyze the impact of different environmental regulations on GTI, and constructing a moderating effect model to study the role of corporate R&D investment and government support in the process of environmental regulations affecting GTI. The results indicate that (1) ER1, ER2, and ER3 can all promote enterprise GTI, and the three environmental regulatory methods have a better synergistic effect. (2) R&D investment has a positive correlation with the relationship between ER2 and GTI, and a negative correlation with ER 3 and ER 1. (3) There are differences in the GTI performance of enterprises in different regions, ownership nature, factor density, and industry types under the influence of environmental regulations. (4) The impact of environmental regulatory policies on corporate GTI is mainly short-term. This study provides a new perspective on how environmental regulations affect corporate GTI, especially in the context of developing countries like China. The research findings emphasize the role of different types of environmental regulations in incentivizing corporate GTI, while also pointing out factors that governments need to consider when formulating environmental policies, such as regional differences and corporate characteristics, which are of great significance for promoting green development of enterprises and achieving broader sustainable development goals.

## 1 Introduction

In recent years, environmental pollution has given rise to a plethora of issues, disrupting the balance of ecosystems and yielding a host of consequences. For example, water pollution has led to a significant mortality rate among aquatic organisms, profoundly impacting the normal functioning of aquatic ecosystems. Furthermore, environmental pollution has inflicted both direct and indirect harm on human health. To illustrate, the harmful substances in air pollution can infiltrate the human respiratory system, giving rise to respiratory diseases and contributing to the proliferation of chronic illnesses.Moreover, environmental pollution has had a noticeable impact on social stability. For instance, water resource pollution and its scarcity have given rise to dissatisfaction and conflict within society. Additionally, this pollution has engendered adverse effects on economic development, including escalated costs associated with environmental management, reduced efficiency in resource utilization, and diminished competitiveness among businesses. This sequence of issues underscores the multifaceted impact of environmental pollution on ecology, health, society, and the economy.

The impact of environmental pollution on the economy is a complex issue involving economic growth, industrial structure, population structure, foreign trade and other aspects. Muhammad Azam [1] used the data of 11 Asian countries from 1990 to 2011 to make an empirical analysis of carbon emissions and economic growth. The results indicated that the deterioration of the environment had a negative impact on economic growth. Therefore, environmental protection policies that reduce carbon emissions should be adopted to control environmental degradation and achieve sustainable economic development. Wei et al [2] found that by transferring backward heavy polluting industries from developed regions to underdeveloped regions to promote the optimization and upgrading of industrial structure, pollution will also be transferred to underdeveloped regions.Population aging and fertility rate increase will lead to environmental degradation.Menz et al [3] studied the relationship between population transition and carbon dioxide emissions in OECD countries. Using panel data for 26 countries from 1990 to 2005, the authors found that increasing population age and fertility would lead to an increase in carbon emissions through a regression of the relationship between population age, birth rate and carbon dioxide emissions. Stahls et al [4] estimated the impact of international trade on forest vegetation biomass and carbon storage in Finland, and the analysis showed that export trade would lead to environmental degradation.

The influence mechanism of environmental pollution and green technology innovation is the research basis of other issues, which have been studied by many people. Environmental pollution has a serious negative impact on human society and ecosystems, and green technology innovation is considered to be one of the key paths to reduce environmental pollution and achieve sustainable development.

There are various views on the relationship between environmental regulation and green innovation technology. According to a school of thought, environmental regulation promotes green technology innovation. Porter [5] argued that appropriately designed environmental regulations can direct firms towards innovation and incentivize them to attain innovation that outweighs compliance costs, thereby enhancing their market competitiveness. When environmental regulation remains stable and highly predictive, it can effectively promote technological innovation and enhance the overall technical indicators (GTI) of enterprises [6]; another point of view is that environmental regulation will increase the production cost of enterprises, squeeze the innovation funds of enterprises to a certainextent, and thus inhibit the green technology innovation behavior of enterprises [7]. The two views take different premises and reach vastly different conclusions, albeit with varying degrees of empirical support. One of the main reasons for this phenomenon is that existing empirical research is commonly hindered by the endogeneity of environmental regulation and pollution control, the difficulty in accurately measuring green technology innovation, and the non-linear effects of environmental regulation [8]. Due to these limitations, it is impossible to accurately assess the impact of environmental restrictions on green technology innovation.

There are different research curves for environmental regulation and green technology innovation. Environmental regulation research curves are generally long and relatively stable. Environmental regulation is mainly reflected in the direct effect on environmental pollution through environmental regulation legislative procedures, legal policies and investment in pollution control [9].They are usually formulated and implemented by the governments to protect the environment by limiting pollution emissions and managing the use of natural resources. The research focus of environmental regulation is mainly on the nature and influence of the environmental problems to be solved. However, with the increasingly prominent environmental problems, the research focus has gradually shifted to the implementation process, the evaluation of the effectiveness of existing regulations and so on. In contrast, the research curve of green innovation technology is more complex and diversified. Green technology innovation is the development and improvement of production technologies to improve resource utilization and reduce environmental pollution, such as developing new energy technologies and improving resource utilization to reduce pollution. In the early stage, the research of green innovation technology mainly focused on the development of new technology and found a feasible solution;at a later period, it mainly focused on improving production technology to improve the level of environmental protection. Although the research curves of the two are different, they complement and interact with each other to jointly promote sustainability.

Environmental problems have perennially presented a puzzle, remaining unresolved.This article mainly answers the following research questions: What are the differences between various environmental regulatory strategies in encouraging green innovation in enterprises? How do different environmental rules affect green technology innovation in different regions and industries? What are the regulatory effects of internal R&D investment and external government support, and how do they affect green innovation in enterprises? What is the time effect of environmental regulations on green innovation in enterprises?Compared with previous research directions, the main contributions of this study are as follows: (1)This paper examines the impact of three environmental regulations on corporate GTI. (2)Using the benchmark regression model and the moderating effect model, the impact of environmental regulation on corporate GTI is discussed. (3)Fully consider the huge differences in ownership, factor quality and other aspects of Chinese enterprises. Based on the study of the relationship between environmental regulation and corporate GTI, this paper provides a theoretical basis fora more rigorous implementation of environmental regulation policies in China, with extensive heterogeneity analysis.(4)This paper distinguishes high-quality innovation from low-quality innovation in the selection of GTI proxy variables, and selects matching variables accordingly, thus reflecting the impact of environmental constraints on corporate GTI in more detail. This strategy is more realistic when considering the autonomy of enterprises ’ creative R&D practice. (5)This paper examines the regulatory impact of R&D investment and government support from both internal and external aspects. In contrast to previous studies, which only examined the relationship between the two from a single perspective, this paper broadens the logical framework. In addition, this paper also examines the impact of environmental legislation on delay.

The following section of this article is arranged as follows: The second section delves into the research literature related to environmental regulation and green technology innovation (GTI) in the business field; section 3 is devoted to theoretical analysis, which improves the framework of the paper and provides theoretical underpinnings for empirical studies; sections 4–6 are the empirical analysis portion of this paper, introducing empirical methods, samples, regression results, and so on. The seventh part is the conclusion of this chapter, and the eighth part is the policy recommendations and future research directions based on the conclusions.

## 2 Literature review

This study focuses on the role of environmental regulations in corporate green technology innovation, with the most relevant reference materials covering research perspectives on environmental regulations and the relationship between environmental regulations and green technology innovation. The following is an overview of existing literature based on these two aspects.

### 2.1 Research on environmental regulation itself

The idea that environmental pollution has strong negative externalities is considered to be a consensus view among academics and policymakers, and as a result, environmental regulation policies have become a hot spot for research.Generally speaking, environmental regulation involves government intervention in the production and operations of companies through administrative measures to achieve the goal of harmonizing environmental protection and sustainable growth.The meaning of environmental regulation is constantly evolving and changing over time. In addition to the rules and regulations implemented by the government through administrative means, it also includes market-based incentive supervision, as well as guidance and regulation of public behavior. This indicates that environmental regulation is not limited to traditional regulatory enforcement, but also includes influencing and guiding individual and organizational environmental behavior through economic incentives and social ideology [10]. Environmental regulation can be divided into three main categories based on its implementation methods: command based, market based, and voluntary. Command based regulation, also known as ER1, relies on mandatory policies implemented by administrative departments. Market based regulation, or ER2, is a method based on market mechanisms and economic incentives. Voluntary regulation, referred to as ER3, relies on public participation and supervision for implementation. This classification reflects the diversity of environmental regulatory strategies aimed at achieving environmental goals through different approaches and methods [11]. Environmental regulations can be classified in various ways, with one common classification being formal regulations and informal regulations. Formal regulations are those based on specific laws or regulations, while informal legal rules are based on non statutory standards or agreements. This classification method highlights the different sources and enforcement methods of environmental regulations, showcasing diverse approaches to environmental protection [12]. Regardless of the type, environmental control measures fundamentally guide enterprises to adjust their production and technological decisions, internalizing their external costs [13]. However, the current definition of environmental regulation is inconsistent, and there is still significant controversy over the methods and intensity of regulation, which has led to different empirical literature substituting regulatory measures. The table below mainly shows the variables of environmental control substitutes selected from existing studies.

In this study, the reason for choosing the proxy variables were as follows:

1. For ER1, according to the literature, the number of regulations and thenumber of administrative penalty cases are used as contingency agents for environmental regulation [14]. These proxy variables can reflect the government ’s regulation of environmental behavior and reveal the intensity and effect of environmental regulation.
2. For ER2, according to the literature, indicators such as pollution discharge fees, industrial pollution control investment, and the number of environmental acceptance projects are used as contingency agents for environmental regulation [15]. These proxy variables can reflect the economic incentives and normative pressures on environmental behavior of companies, and facilitate the implementation of more environmentally friendly measures by companies.
3. For ER3, according to the literature, the received environmental information, the number of visits and the real number of people involved in environmental protection are used as contingency agents for environmental supervision [16]. These proxy variables can reflect the degree of public concern andparticipation in environmental issues, and have an impact on the implementation of environmental regulatory policies.
4. For Formal ER, according to the literature, indicators such as pollutant emission removal rates, pollution control investment rates, and pollution are used as contingency agents for environmental regulation [17]. These proxy variables can effectively drive the adjustment of industrial structure.
5. For informal formal ER, according to the literature, contingency agents for environmental regulation are used with indicators such as environmental awareness, per capita income, and education level [18]. These proxy variables can enhance people’s attention and willingness to act on environmental issues, thus facilitating the effective implementation of environmental regulation.

Given the diversity of environmental regulation policymaking in China.In this article, the stimulus effect of government environmental laws on enterprise GTI is emphasized by us. In the empirical analysis section of this article, three policies will be combined with a separate policy to compare their impact on green environmental protection with ER1, ER2, and ER3.

### 2.2 Research on the relationship between environmental regulation and green technology innovation

Porter followed the logical framework of "endogenous growth" and for the first time combined technological innovation and environmental regulation into a unified theoretical framework, and based on this, proposed the Porter hypothesis. On this basis, many studies have been conducted on this hypothesis. Among these methods, the most direct approach to testing Porter’s hypothesis is by promoting green technological innovation through environmental regulations. However, the research findings regarding this approach have generated controversy. On the one hand, When studying the relationship between environmental regulation, innovation, and competitiveness, the perspective of maximizing profits should always be regarded as the main driving force for corporate strategic decision-making. This viewpoint holds a central position in any business decision-making consideration. Therefore, it is often unrealistic to believe that compliance costs or underestimating profit maximization factors can be ignored when companies decide whether to comply with specific regulatory stimulus measures or take proactive measures in the environment [19]. It was found that environmental regulations based on taxes and fees significantly hinder technological innovation by enterprises. Environmental regulations lead to an increase in production costs for enterprises, limiting their ability to engage in technological innovation [20]. In contrast, some studies suggest that environmental regulation does indeed have the compensation effect for innovation proposed by Porter. Environmental regulations have a significant positive impact on green technology innovation in enterprises. Environmental regulations lead to increased taxes fees, forcing large and medium-sized enterprises to engage in green technology innovation to reduce energy loss [21]. The starting point of this study is not to deny the existence of compliance costs, but to emphasize that innovation compensation exceeds compliance costs.Wang [22] examines the impact of environmental regulation (pollution fees) on green technology innovation from the perspective of compliance costs. Intended to demonstrate that innovation compensation exceeds compliance costs. The above two perspectives both explore the spillover effects of green technology from a linear relationship perspective, but do not consider the impact of environmental regulation levels on the spillover effects of green technology. From the above two aspects, the effect of environmental constraints on green technology innovation is linear, which means that in green technology innovation, the effect of environmental constraints on green technology innovation is marginal and fixed. Although this assumption is theoretically valid, it overlooks the possibility of the marginal effect limit of environmental laws on green technology progress, thus proposing research on the appropriate intensity of green technology progress. Based on the background of heterogeneous industries, Shen [23] identified the optimal degree of industrial regulation in China through non-linear analysis of environmental regulation and efficiency.Li Ling and Tao Feng [24] found that in China’s medium and low pollution industries, the impact of environmental regulation on green TFP and the impact of technological innovation on technological efficiency exhibit a U-shaped curve, and their speed of breaking through the U-shaped inflection point is faster than that of productivity and technological innovation. Bing et al [25] pointed out that different levels of environmental regulations have different effects on corporate green innovation.Tang et al [26] believe that compared to the other two types of environmental regulations, ER1, as the initial stage of green industry development, plays a much greater role in promoting innovation in the green industry. ER2 and ER3 experience stable growth during the high-income period of the economy,while ER1 serves as the foundation. Environmental restrictions have an inverted U-shaped impact mechanism on GTI [27] or a U-shaped impact mechanism [28], according to other research findings.

In conclusion, the existing literature has examined the relationship between environmental regulation and Green Technological Innovation (GTI) from a multitude of perspectives. While this exploration has yielded valuable insights, the findings remain a subject of contention. In this research, it is claimed that the following are the primary causes of the dispute in existing studies: 1.The game between the "follow cost" effect and the "innovation compensation" effect determines the inhibitory or promoting effect of environmental constraints on green technology innovation in enterprises. This is related to the way and intensity of environmental regulation. Different countries are at different economic stages in the country study, which may lead to variance in the empirical study outcomes. In terms of empirical variable selection, there is variability in the selection of environmental rules and GTIs as proxies, resulting in inconsistencies in the results, which is essentially an under-robustness of empirical findings. The main reasons for this are the endogeneity of environmental and pollution control, the difficulty in accurately evaluating green technology innovation, and the nonlinear effects of environmental regulation. Additionally, existing literature has primarily conducted empirical analyses at the regional or industry level, with a focus on industrial, manufacturing, and heavily polluting industries. However, there is limited research addressing the industry as a whole from a macroeconomic perspective, which may result in a degree of generality in the findings. Secondly, existing studies have mostly explored the mechanism of heterogeneous environmental standards on green innovation from the perspectives of both internal and external enterprises. yet it has overlooked the intricate interactions between government and businesses.This technique may not accurately portray the true situation, particularly when considering government incentives in many nations to encourage the development of green technology by businesses. Thus, this paper delves into the impact of diverse environmental regulations on firm Green Technological Innovation (GTI) and dissects its mechanisms from both internal and external coupling perspectives, with China’s listed A-share companies as the subject of study.

## 3 Theoretical analysis

This project takes the spillover effect of green technology as the starting point, and explores the mechanism of environmental regulations on the spillover effect of green technology from the perspective of internal and external coupling. Internal and external coupling refers to the process in which internal and external factors work together on technological innovation. On this basis, A theoretical analysis of the effects and mechanisms of ER1, ER2, and ER3 on corporate green innovation behavior was established.

On this basis, a theoretical analysis framework is constructed from two levels: the mechanism of environmental regulation on green innovation behavior, and the mechanism of enterprise investment and government subsidies in green innovation. Command oriented ER constrains corporate environmental behavior through mandatory actions of laws and regulations; Market oriented ER adopts environmental tax and emissions trading plans to guide enterprises in reducing pollutant emissions; Voluntary ER relies on third-party supervision and public supervision to encourage enterprises to fulfil their environmental responsibilities. Enterprises have implemented production technology reforms in line with the requirements of these environmental regulations to reduce energy consumption and improve productivity. At the same time, with the R&D investment from internal enterprises and the support from external governments, enterprises have introduced environmental protection technologies to promote green transformation and sustainable development of enterprises.

### 3.1 Environmental regulation and enterprise GTI

The "innovation compensation" effect and the "follow cost" effect are important mechanisms of environmental regulation on green innovation behavior, and they are the mechanisms of environmental regulation on green innovation behavior. However, at the micro level, different environmental control methods have different impacts on corporate green innovation behavior through different operating mechanisms. In this section, the theoretical impact of ER1, ER2, and ER3 on firm GTI is examined.

#### 3.1.1 The influence of command-based environmental regulation (ER1) on enterprise GTI

In order to achieve success, command based environmental regulation (ER1) is an environmental regulation method that is enforced by the government through mandatory means, relying on laws and regulations formulated by various levels of government. In China, ER1 is usually divided into national and provincial policies and regulations. At the national level, the National People’s Congress has reviewed and formulated China’s environmental protection legislation and regulations, which have global and fundamental guiding principles. The new Environmental Protection Law formulated and implemented by China in 2015 is a typical example of environmental regulations.These regulations demonstrate the Chinese government’s stricter punishment measures for corporate illegal behavior, increasing the costs incurred by companies due to pollution. Encourage enterprises to increase investment in research and development, accelerate technological innovation, and compensate for the increase in environmental costs [29]. ER1 refers to provincial-level environmental policies and regulations formulated by each province based on national environmental laws and regulations as well as local conditions. Under this environmental regulatory framework, provincial governments formulate specific environmental policies and regulations applicable to the province based on national regulations and local conditions.Due to the differences in economic development and industrial structure between provinces, this model may better reflect the ac Due to the differences in economic development and industrial structure between provinces, this model may better reflect the actual situation of the province, and it is fundamentally consistent with national laws and regulations. tual situation of the province, and it is fundamentally consistent with national laws and regulations.The biggest disadvantage of ER1 is its lack of flexibility and high execution costs. ER1 has mandatory and efficient characteristics, which can achieve short-term optimization of industrial structure.For enterprises with lower levels of economic development, ER1 will inhibit the development of green technology innovation. For enterprises with average economic development level, the impact of ER1 on green technology innovation and industrial structure is relatively weak. Only when the economic development level of enterprises tends to be higher, will ER1 significantly promote green technology innovation and industrial structure upgrading [30].

#### 3.1.2 The influence of market-based environmental regulation (ER2) on enterprise GTI

Market-based environmental regulation (ER2) is another type of government-implemented environmental regulation. In contrast to the mandatory law of ER1, ER2 focuses on the government guiding the polluting behavior of firms through market-based means, and its key features are non-compulsory and traceable. The environmental tax and emissions trading plan is a common ER2 case. China launched an environmental tax system in 2018.As a statutory tax, environmental protection tax demonstrates its independence and reasonable tax system. Compared to mandatory environmental regulation, environmental protection taxes are more precise and give local governments more autonomy in setting tax rates. This allows tax rates to be flexibly adjusted based on the overall operating conditions of the enterprise, avoiding low tax standards that result in the cost of pollutant emissions being much lower than the cost of innovation inputs, thereby failing to incentivize innovation. On the contrary, high tax standards may cause companies to move away from the local area under heavy economic burdens, resulting in a loss of tax base and negative impacts on local taxation and economic development. The implementation of environmental taxes helps stimulate the innovation drive of enterprises and significantly promotes the input and output of technological innovation. This comprehensive tax system helps to balance the relationship between environmental protection and economic development, and enhances the environmental protection and innovation awareness of enterprises [31]. Emissions trading is another common form of ER2. Laffont and Tirole [32] revealed that emission permit can stimulate companies to invest excessively in eco-friendly innovation, aiming to bypass the monetary burden of reducing social welfare. In the long run, it is crucial in guiding enterprises to invest in GTI. Generally speaking, in situations where environmental protection intensity is not high, companies will prioritize the development of environmental protection technology. In other words, in order to comply with environmental regulations, they will purchase pollution discharge permits or pay taxes. When the intensity of environmental pollution increases, companies will invest more in green technology innovation to obtain long-term benefits.

#### 3.1.3 The influence of voluntary environmental regulation (ER3) on enterprise GTI

In terms of environmental regulation, voluntary environmental regulation (ER3) is the highest level of public environmental awareness. Compared to ER1 and ER2, voluntary environmental regulations focus on setting goals, strategies, and development guidelines to improve corporate environmental performance, rather than regulating specific methods for achieving these goals, which gives companies maximum flexibility [33]. There are currently two types of environmental self-discipline mechanisms in China, one is a third-party environmental certification system, and the other is a social supervision system. Empirical evidence suggests that adopting third-party environmental supervision can improve a company’s environmental performance, including reducing pollutant emissions [34]. These works have established a good reputation for the enterprise. This will help promote the development of green technology innovation. The emergence of the Internet has heightened public scrutiny, leading to the real-time recording and disclosure of firms’ pollution conditions.Enterprises may face pressure from consumers, investors, the public/community, employees, and contractors/suppliers to take measures to improve environmental management [35]. As a result, enterprises are compelled to respond to both administrative and public pressure. In terms of environmental regulation, voluntary environmental regulation (ER3) marks the peak of public environmental awareness. Compared to ER1 and ER2, the implementation of this policy is relatively small, and its level of implementation mainly depends on the public and economic entities’ understanding of environmental protection. From a long-term perspective, green industry funds are the best choice for enterprises. Overall, ER3 tools can better stimulate the environmental enthusiasm of the public and enterprises, effectively reducing government enforcement and regulatory costs, and stimulating enterprises to actively participate in green technology innovation [36].

#### 3.1.4 Case study on the impact of environmental regulations on enterprise GTI

*3*.*1*.*4*.*1 Case Study on the Impact of ER1 on Enterprise GTI*. In the case of a paper-making business, it was under great pressure more than a decade ago, owing to the excessive discharge of sewage and the lack of sewage treatment capacity. Under the strict ER1, the enterprise began to control pollution in 1993.Following 56 years of environmental management, in 1999, the largest oxidation pond for papermaking pollution control in China was constructed. After treatment, the chemical oxygen demand (COD) of the sewage decreased to 350mg / L, which was one year ahead of the national requirement that the sewage must reach the standard discharge (COD content 350mg / L) by the end of 2000.However, due to excessive environmental protection, costs have increased and the business has remained in a low-profit state, with no significant improvement in economic benefits. ER1 may not have an obvious effect on motivating firms to develop GTI [37].

*3*.*1*.*4*.*2 Case Study on the Impact of ER2 on Enterprise GTI*. Taking Baoshan Iron and Steel Co., Ltd. as an example, the company is located in the high pollution and high energy consumption steel industry, and has always been highly valued by the national environmental protection department. Therefore, the driving effect of environmental regulations on its green technology innovation is very obvious. Command controlled environmental regulations impose institutional constraints on enterprises by stipulating mandatory environmental standards, imposing penalties for environmental pollution, and even ordering production stoppages. In order to ensure normal production and operation, Baosteel has had to transform its production process and carry out green technology innovation. The market incentive type mainly focuses on guiding and encouraging, compensating enterprises for green technology innovation behavior through tax incentives and environmental subsidies, and incentivizing enterprises to carry out green technology innovation. In order to solve the problem of environmental pollution and balance the relationship between enterprise production and operation and national environmental regulations, Baosteel has significantly increased its investment in environmental protection. Since 2012, it has been a comprehensive improvement stage of China’s environmental regulation policy system. With the introduction of a large number of environmental regulatory policies, Baosteel’s investment in environmental protection has fluctuated from 3.977 billion yuan in 2016 to 12.16 billion yuan, an increase of 2.06 times [38].

*3*.*1*.*4*.*3 Case Study on the Impact of ER3 on Enterprise GTI*. Taking Huatai Company as an example, since 2007, Huatai has unswervingly carried out a series of work in accordance with the requirements of the environmental management system, taking the environmental policy as the basic criterion, starting from the environmental elements, taking the environmental objectives, indicators and programs as the focus of work, and achieved remarkable environmental performance. Huatai has added facilities to recover biogas, which is produced by anaerobic digestion and used for thermal power generation. In 2007, a total of 16% of biogas was produced, equivalent to 17,000 tons of standard coal, creating an efficiency of 6.78 million yuan.By improving processes, retrofitting equipment and other measures, enhancing energy conservation and emission reduction assessments, and controlling energy consumption, the company achieved annual power savings of 100 million kWh and steam savings of 220,000 m2 in 2007. The implementation of voluntary environmental regulation policies is precisely responsible for enterprises achieving multiple outcomes. This includes not only the reduction of pollutant emissions and an increase in income but also the objective promotion of technological advancement and innovation within these enterprises [39].

### 3.2 The moderating role of enterprise R&D investment and government support

In the preceding article, various kinds of environmental regulations that may force or guide enterprises to engage in GTI activities to some extent were examined.But the specific microscopic mechanism is still unclear. On this basis, from the perspectives of enterprise R&D investment and government support, this paper explores the micro mechanism of environmental legislation on green technology innovation.This technique was chosen because financing is the most important issue for organizations pursuing GTI as a research and development activity.

The technological innovation at the enterprise level is mainly influenced by R&D management drivers. Research has shown that R&D investment is an effective way to improve the environmental technology innovation capability of enterprises [40]. In the context of government enforcement of environmental regulations, enterprises have two options: increasing overall R&D investment and adjusting the structure of R&D investment.First, environmental rules encourage companies to increase R&D spending in order to take sustainability into account, and this upswing in total R&D spending consistently correlates with a rise in green R&D investments. Secondly, when facing environmental regulations, even if R&D investment remains unchanged, companies will adjust their own investment to encourage them to increase investment in green technology innovation; Meanwhile, increasing investment in green research and development can also enhance the awareness of enterprise researchers towards green technological innovation [41]. In addition, by increasing research and development investment, enterprises can implement green and low-carbon development strategies, improve pollution control levels, and minimize the source of pollution. This not only reflects the company’s high social responsibility, but also enhances its brand image. This can also help alleviate economic development and environmental issues.

Having established environmental legislation, government subsidies, tax incentives and other measures may have a more unique benefit in reducing the problem of companies underinvesting in green R&D. Corporate GTI is known for its high investment and high risk, and risk averse enterprises may not be interested or participate in GTI at all. From this perspective, short-term environmental constraints will only encourage companies to use carbon emissions trading to reduce production scale, rather than carbon emissions reduction. Currently, government assistance can to some extent bridge the gap between social welfare and personal well-being, reduce market failures in the green industry, and enhance the enthusiasm of enterprises for the development of the green industry [42]. On the other hand, obtaining government funding is a message conveyed by external investors that these enterprises typically possess strong innovation capabilities and development potential.These businesses hold a government-recognized status, which makes it easier for them to earn the trust of external investors. As a result, they can broaden their sources of funding, reduce funding expenses, enhance their negotiation leverage, bolster the risk-reduction capacity within the green innovation supply chain, and ultimately improve their Green Technological Innovation (GTI) capabilities.

## 4 Research design

### 4.1 Variable selection

#### 4.1.1 Dependent variable

GTI Enterprise. In this study, the green utility model and invention patents were chosen as stand-ins for the GTI of businesses.This is the so-called "green invention", which has strong innovation and difficult to achieve characteristics, and can stimulate more research and development investment.Comparing the regression results for these two variables reveals both the propensity of businesses subject to environmental restrictions to be innovative and the effect of environmental rules on the GTI of a business.In order to ensure that innovations that have been applied in practice but have not yet received patent approval are not overlooked.Meanwhile, two indicators, the number of green invention patent applications and the number of green utility models, are selected to characterize the stability of the system. Following the method suggested by Fang and Shao [43], In this work, the comprehensive index approach is used to calculate the three different environmental rules mentioned above for each indicator.The precise calculation procedure is as follows. The corresponding data could be seen the the Supporting Information.

#### 4.1.2 Independent variables

On this basis Three factors, namely ER1, ER2, and ER3, were selected to characterize the intensity of environmental regulation. (1) ER1: This study used the number of environmental protection legislation in each province and city, including the number of administrative penalty cases accepted, the number of environmental supervisors, and the amount of funding for environmental protection facility construction [44]. (2) ER2: This is measured from four aspects: pollution tax (environmental tax), the proportion of environmental expenditure in local fiscal expenditure, the proportion of environmental infrastructure investment in regional GDP, and the proportion of pollution control investment in regional GDP [45,46] (3) ER3: This article selects variable factors for quantitative evaluation of ER3, such as credibility, frequency of environmental inspections, proposals from the Two Sessions, automatic monitoring of key pollution sources, and nature reserves. Table 1 showed the specific description of independent variables.

**Table 1:** Specific description of independent variables.

Variable	Variable Symbol	Variable action	Variable basis
Command based environmental regulations	ER1	Impact corporate GTI through government regulations	National laws and regulations related to environmental protection; Administrative regulations and rules formulated by administrative departments in accordance with national laws and regulations; Refer to relevant environmental standards to control environmental quality.
Market oriented environmental regulations	ER2	Impact on corporate GTI through increased environmental taxes and emissions trading	Using emission permits, emissions trading, etc. as economic tools; Set prices or taxes for pollutant emissions and encourage enterprises to adopt environmentally friendly behaviors; Establish a reward mechanism to encourage enterprises to exceed environmental goals and improve environmental performance.
Voluntary environmental regulations	ER3	Impact GTI of enterprises through third-party environmental certification and public supervision	Voluntary environmental management system based on voluntary participation of enterprises; Environmental standards voluntarily adopted by enterprises; Enterprises voluntarily disclose environmental data and report environmental performance.

Standardize the individual indicators:

$${UE}_{ij}^{s}=\frac{{UE}_{ij}-Min({UE}_{j})}{Max({UE}_{j})-Min({UE}_{j})}$$ (1)

Where, ${UE}_{ij}^{s}$ represents the standardized value of index *j*, *UE*_*ij*_ represents the initial value of index *j* in province *i*, *Min*(*UE*_*j*_) and *Max*(*UE*_*j*_) respectively represents the minimum and maximum value of index *j* in all province, *i* represent each province, *i* = 1,2, …,30; *j* represents the indicators of each environmental regulation.

In order to eliminate the differences between similar indicators in various regions, the adjustment coefficient is calculated as follows:

$$W_{j}=\frac{{UE}_{ij}}{{UE}_{j}}$$ (2)

Among them, *W*_*j*_ represents the adjustment coefficient of the *j* index, and *UE*_*j*_ represents the average value of the j index in the sample.

Calculate the comprehensive index of ER1, ER2 and ER3 according to the following formulas:

$${ER}_{it}=\frac{1}{n}\sum_{i=1}^{j}\left(W_{j}\times {UE}_{ij}^{s}\right)$$ (3)

*ER*_*it*_ represents the comprehensive index of environmental regulation in province i in period t.

#### 4.1.3 Moderator variables

This article uses R&D investment and government support as mediating variables. Financial investment is the most crucial aspect in the process of research and innovation. On this basis, this project found through empirical research that there is a significant correlation between the company’s internal financing capacity and external financing capacity in terms of environmental regulation and green innovation performance. Under the framework of green innovation capability, internal financing capability of enterprises is an important component of their R&D activities. External financial capability refers to the ability of enterprises to receive support from the government for green innovation. This study selects these two factors as mediating variables to examine their impact on environmental regulation and corporate green innovation behavior. This article quantitatively analyzes the government’s support for research and development investment, enterprise investment, and other aspects based on the financial subsidies received by enterprises.

#### 4.1.4 Control variables

In this paper, The following control factors, which are expected to affect firm GTI at both firm and region levels, are selected. Company size, capital intensity, returns, board governance, and company maturity are all selected. The size of each province is determined by factors such as the level of openness, marketization, regional economic development, and innovation environment. Therefore, this project intends to select these two variables as mediating variables to examine their impact on the green innovation behavior of enterprises. This article uses the company’s R&D investment as an indicator to evaluate R&D investment, and the government’s evaluation of R&D investment is measured by the government subsidies received by enterprises.

### 4.2 Data sources

The research samples of this paper are taken from the data of China ’s A-share listed companies On the basis of collecting the original data, the original data is processed: (1) Remove ST (listed companies with two consecutive years of losses) and * ST (listed companies with three consecutive years of losses) enterprises. (2) Due to the lack of data, remove the samples of Hong Kong, Macao, Taiwan and Tibet. (3) Delete company samples with severely missing data, which cannot be obtained and affects the operation of the software. (4) If the company lacks a small amount of data,After being supplemented through public databases, there is no significant difference. survey questionnaire data, or speculation based on other variables. (5) Adding 1 to the green patent data, and taking the log. As a result, the total sample size was3930observations.From an econometric point of view, the sample size chosen for this paper has been able to be compelling in a certain statistical sense.

In order to ensure the adequacy of the collected data, our data comes from: (1) Matching the company name with the China Research Data Service Platform (CNRDS) to obtain green patent data. (2) The company ’s name is linked to the accounting research database(CSMAR) and CNINFO website (www.CNINFO.com.cn) to obtain relevant financial data. (3) ’ China Statistical Yearbook ’, ’ China Environment Statistical Yearbook ’, ’ China Science and Technology Statistical Yearbook ’, ’ China Industry Statistical Yearbook ’ and ’ China Energy Statistical Yearbook ’.

### 4.3 Model construction

(1) Benchmark regression model

The benchmark regression in this paper is to use enterprise GTI to regression ER1, ER2 and ER3 respectively. The detailed model is shown in formula (4).


$${GTI}_{it}=\alpha_{0}+\alpha_{1}{ER}_{it}+\alpha_{2}{Con}_{it}+\mu_{i}+\varepsilon_{it}$$ (4)

Among them: *GTI*_*it*_ represents the green technological innovation level of the i company in the t period, which is divided into green invention patents (GTI1) and green utility model patents (GTI2); *ER*_*it*_ represents the environmental regulations of the province where the i company is located during the t period, As mentioned above, this variable actually contains three indicators, ER1, ER2 and ER3. For the purpose of simplification, the three variables are marked with the same variable symbol in formula (4); *Con*_*it*_ represents a set of control variables, *μ*_*i*_ represents an individual fixed effect, and *ε*_*it*_ represents a random interference item.

(2) Moderating effect model

In order to test the moderating effect of R&D investment and government support on the relationship between environmental regulation and corporate GTI, this paper constructs two moderating effect models for regression. The concrete model is shown as Eq (5) and Eq (6).


$${GTI}_{it}=\beta_{0}+\beta_{1}{ER}_{it}+\beta_{2}{RD}_{it}+\beta_{3}{{ER}_{it}\times RD}_{it}+\beta_{4}{Con}_{it}+\mu_{i}+\varepsilon_{it}$$ (5)

$${GTI}_{it}=\theta_{0}+\theta_{1}{ER}_{it}+\theta_{2}{Sub}_{it}+\theta_{3}{{ER}_{it}\times Sub}_{it}+\theta_{4}{Con}_{it}+\mu_{i}+\varepsilon_{it}$$ (6)

Among them: *RD*_*it*_ represents the level of R&D investment of enterprise i in period t; *Sub*_*it*_ represents the government support received by enterprise i in period t; other variables are explained as above.

## 5 Empirical results and analysis

### 5.1 Descriptive statistical analysis

Table 2 shows the descriptive statistics of the variables. These variables include GTI1, GTI2, ER1, ER2, ER3, RD, Sub, InSize, Den, Pro, BG, Age, FDI, ML, GDP, and Atm. For the baseline regression of this article, each variable had 3930 observations. From an average perspective, the average value of GTI1 was 1.5223, GTI2 was 1.2015, ER1 was 0.6415, ER2 was 0.8263, ER3 was 0.7415, RD was 3.8456, Sub was 1.3263, InSize was 8.1253, Den was 2.6123, Pro was 0.6552, BG was 0.2012, Age was 10.362, FDI was 0.0326, ML was 0.3115, the average GDP was 0.0003, and the average Atm was 0.5163. For these variables, the standard deviation ranged from 0.003 to 12.3625. The minimum and maximum values provided the range of values for each variable. For example, the minimum value of GTI1 was 0.0002 and the maximum value was 8.3623. The minimum value of ER1 was 0.0003 and the maximum value was 3.7485. The minimum value of RD was 0.0003 and the maximum value was 224.1256. In general, the explained variables and the environmental regulation intensity variables (ER1, ER2, ER3) had relatively wide ranges, reflecting differences in environmental regulation policies across provinces within China, and pointed to the need for region-based heterogeneity analysis in the paper that follows.

**Table 2:** Variable descriptive statistics.

Variables	count	Mean	SD	Min	Max
GTI_1_	3930	1.5223	1.5263	0.0002	8.3623
GTI_2_	3930	1.2015	1.0013	0.0001	6.4159
ER_1_	3930	0.6415	0.9956	0.0003	3.7485
ER_2_	3930	0.8263	0.3245	0.0015	3.6592
ER_3_	3930	0.7415	0.6516	0.0086	5.1125
RD	3930	3.8456	12.3625	0.0003	224.1256
Sub	3930	1.3263	2.8459	0.0004	53.2369
InSize	3930	8.1253	1.5216	3.7856	19.2415
Den	3930	2.6123	2.5523	0.2036	85.1263
Pro	3930	0.6552	0.0662	-1.0012	0.6411
BG	3930	0.2012	0.0849	0.3412	1.0445
Age	3930	10.362	5.6632	1.0236	31.326
FDI	3930	0.0326	0.0487	0.0086	0.9985
ML	3930	0.3115	0.5263	0.0816	0.9826
GDP	3930	0.0003	0.0003	0.0002	0.0053
Atm	3930	0.5163	0.1256	0.0526	0.9895

### 5.2 Benchmark regression results

In this paper, a fixed-effect model has been chosen for regression analysis. It incorporates controls at both the individual and time dimensions, which can help alleviate endogeneity issues arising from individual characteristics and time trends to a certain extent. The benchmark regression results are shown in Table 3. The regression findings with green innovation patents as the explanatory variables are shown in columns (1) through (4). Columns (5) through (8) of the green utility model are interpreted variables. Columns (1) and (5) show that ER1 has a good impact on both green invention patents and green utility model patents, indicating that ER1 may help the firm’s GTI. Each unit increase in ER1 strength was associated with a 0.084 and 0.096 unit increase in the level of green invention and utility model patents, respectively.However, ER1 is not significant for green invention patents, while ER1 passes the 5% significance level test for green utility model patents.Based on the previously mentioned findings, ER1, the most widely adopted regulatory approach in China, has been observed to play a significant role in driving efforts for the development of Green Technological Innovation (GTI).(2) and (6) demonstrate that ER2 has distinct effects on the green invention patent and the green utility model patent. Although the regression results indicate that the coefficient of influence of ER2 on green invention patents is 0.236 and has a beneficial effect, this finding is not statistically significant.The green utility model patent has also been severely damaged by ER2. For every unit increase in ER2 intensity, the patents for green utility models decrease by 0.299 units and pass the 10% significance level The research results show that China’s current environmental protection system still has a market-oriented environmental protection model, which restricts green and low-carbon development. From columns 3 and 7, it can be seen that ER3 has a significant impact on both green and green utility models, although in the opposite direction. The study found that the impact of ER3 index on green innovation patents is significant at 5%, and for every 1 unit increase in ER3 index, it decreases by 0.078.On the other hand, ER3 greatly boosted the green utility model patent at the5% significance level, with each unit increase in ER3 strength increasing the patent on the green utility model by 0.245.Research has found that companies are more inclined to adopt green utility model patents rather than green invention patents when facing voluntary environmental regulations. Even when using green utility models for patent creation, there is still an issue of excessive reliance on green invention patent resources. Alternatively, this result could be due to the tendency of firms to engage in short-term regulatory behavior such as end management in the face of public and media scrutiny, which not only wastes firm resources but also crowd out investment in green invention patenting. Introduce three different environmental regulation intensity variables into the regression analysis at the same time, and provide them in columns 4 and 8, respectively. Under the combined effect of the three adjustment methods, the regression coefficients of all three adjustment methods are relatively large. Using the green invention patent model as the explanatory variable, ER1 is 0.035, ER2 is 0.458, and ER3 is 0.336, respectively, and all are significant at the 5% significance level.Using the green utility model patent model as the explanatory variable, the regression results for ER1, ER2, and ER3 were 0.066, 0.699, and 0.563, respectively, and were significant at the 10% significance level. Furthermore, with the exception of ER1, the regression findings of the other two environmental regulation approaches outperform the regression results of the model with simply themselves as explanatory variables.These findings indicate that the interaction of the three environmental policies is crucial to the green technology innovation of enterprises. Considering the impact of each environmental regulation approach on green invention patents and green utility model patents alone does not stand out, and considering their interplay at the same time allows for a better understanding and optimization of the effects of environmental regulation policies.This policy interaction can increase the quantity and quality of green invention patents and green utility model patents, thereby promoting environmental protection and the development of enterprise GTI.

**Table 3:** Benchmark regression results of environmental regulations and GTI.

Variables	green invention patent				green model patent			
	(1)	(2)	(3)	(4)	(5)	(6)	(7)	(8)
ER_1_	0.084 (2.42)			0.035** (2.96)	0.096** (2.82)			0.066* (3.45)
ER_2_		0.236 (3.41)		0.458** (5.29)		-0.299* (-3.06)		0.699* (5.85)
ER_3_			-.0.078** (-1.48)	0.336** (3.41)			0.245** (0.856)	0.563* (-4.96)
InSize	0.412** (9.03)	0.293* (18.62)	0.426** (10.362)	0.322* (8.02)	0.412** (9.22)	0.549** (8.02)	0.411** (12.36)	0.516* (12.36)
Den	0.085 (3.02)	0.075 (2.58)	0.052 (1.96)	0.026 (1.95)	0.054 (2.36)	0.056 (2.03)	0.054 (2.84)	0.052 (1.99)
Pro	0.599 (0.63)	0.265 (0.81)	0.632 (0.45)	0.159 (0.43)	0.085 (0.45)	0.046 (0.36)	0.055 (0.22)	0.039 (0.05)
BG	0.685*** (1.92)	0.636 (1.85)	0.846 (1.71)	0.612 (1.43)	0.361 (0.96)	0.258 (0.56)	0.236 (0.39)	0.242 (0.53)
Age	0.812** (13.26)	0.312** (12.36)	0.183** (17.13)	0.145** (12.36)	0.075*** (7.03)	0.075* (6.95)	0.084* (8.11)	0.086* (9.03)
FDI	0.312 (0.66)	0.765 (0.21)	0.846** (1.85)	0.944 (1.75)	1.263** (2.03)	1.849** (2.22)	2.956* (4.12)	2.752* (3.81)
ML	0.645 (0.95)	-1.336** (-3.46)	-1.169** (-4.42)	-1.558* (-4.23)	-1.415** (-3.85)	-1.845* (-4.03)	-1.762* (-4.23)	-1.886** (-5.45)
GDP	92.36 (0.94)	-42.16 (-0.42)	29.13 (0.82)	-125.629 (-1.23)	-590.23* (-3.26)	-696.13* (-6.23)	-711.23* (-4.12)	-892.36* (-5.12)
Atm	-0.849** (-4.72)	-0.685** (-3.956)	-0.796** (-7.885)	-0.546** (-3.49)	-0.849** (-4.42)	-0.884* (-4.23)	-0.623* (-4.22)	-0.684** (-4.42)
-cons	-2.112** (-8.16)	-2.748** (-9.12)	-2.236** (-10.22)	-2.362** (-9.42)	-1.996* (-6.23)	-1.845* (-6.26)	-1.955* (-4.96)	-1.845* (-4.96)
N	3930	3930	3930	3930	3930	3930	3930	3930
R^2^	0.312	0.285	0.452	0.336	0.062	0.085	0.071	0.053

In addition, this project will also study the impact of environmental control policies on green invention patents and green practical new technologies, pointing out that environmental control policies have a higher level of support for green invention patents and green practical new technologies. Faced with environmental regulation, enterprises tend to adopt green and low-carbon technologies with low technological content rather than innovative benefits, which is consistent with the reality of most Chinese enterprises, especially small and medium-sized enterprises.

### 5.3 Robustness test

Outliers are cleaned here using Winsorize processing. Based on this, the robustness test is run using one of three methods:

1. Replace the explanatory variable with the number of green patent applications as a proxy for GTI. The number of green patent applications is used to replace the original explanatory variables and check whether the results are consistent with the benchmark regression resultsin the robustness test. This can verify whether the impact of green patent applications on GTI is robust.
2. Remove control variables at the enterprise level. In the benchmark regression, there are control variables at the enterprise level, such as enterprise scale, capital structure and so on. In the robustness test, these enterprise-level control variables are removed from the model to verify whether the results are still robust.
3. Add new control variables. In this robustness test, considering that the differences in education and industrial structure in China ’s provinces may have an impact on the GTI of enterprises, these two factors are added as control variables in the test. The industrial structure coefficient (Str) is calculated by the practical Eq (7), and these two factors are added and introduced into the model to verify whether the results are still robust.

$$Str=\sum_{i=1}^{3}i\frac{Y_{it}}{Y_{t}}$$ (7)

where $\frac{Y_{it}}{Y_{t}}$ represents the proportion of the added value of the i industry (i = 1, 2, 3) in year t to the total output value.

Regression results in the total number of patent applications. The regression coefficient of variable ER1 was 0.811, but not significant. The regression coefficient of variable ER3 was -5.112, which passed the 10% significance level test.

Regression results after removing control variables at the enterprise level. The variable ER1 had a positive impact on the number of green invention patents, with a regression coefficient of 0.042, passing the significance level test of 5%. The variable ER1 also had a positive impact on the number of green utility model patents, with a regression coefficient of 0.085, passing the 10% significance level test. The coefficient of ER3 for green invention patents was -0.012, and was significantly negative at the 5% level. The coefficient of ER3 for green utility model patents was -0.136, and was significantly negative at the 5% level.

Regarding the regression results while simultaneously increasing education levels and industrial structure. Attention was still paid to the number of green invention patents and green utility model patents. The variable ER1 had a positive impact on green utility model patents, with a regression coefficient of 0.045, passing the 10% significance level test. The coefficient of ER2 for green invention patents was 0.212 and was significantly positive at the 10% level. The coefficient of ER2 for green utility model patents was 0.143, and was significantly positive at the 5% level. The coefficient of ER3 for green utility model patents was -0.146, and was significantly negative at the 5% level.

Table 4 showed the robustness test regression results. The aforementioned test findings are essentially compatible with the benchmark regression results, showing that the study’s conclusions are sound.

**Table 4:** Robustness test regression results.

Total number of patent applications	Variables	Total number of patent applications					
		(1)		(2)		(3)	
	ER_1_	0.811 (0.56)					
	ER_2_			13.526 (6.88)			
	ER_3_					-5.112* (-2.43)	
	Con	YES		YES		YES	
	N	3930		3930		3930	
	R^2^	0.035		0.055		0.042	
Remove corporate-level control variables	Variables		green invention patent		green utility model patent		
		(1)	(2)	(3)	(4)	(5)	(6)
	ER_1_	0.042** (2.03)			0.085* (3.42)		
	ER_2_		0.042 (0.88)			0.066 (1.423)	
	ER_3_			− 0.012** (-2.31)			− 0.136** (-4.88)
	Con	YES	YES	YES	YES	YES	YES
	N	3930	3930	3930	3930	3930	3930
	R^2^	0.312	0.355	0.356	0.055	0.057	0.042
At the same time increase the level of education and industrial structure	Variables	green invention patent			green utility model patent		
		(1)	(2)	(3)	(4)	(5)	(6)
	ER_1_	0.053 (2.42)			0.045** (3.45)		
	ER_2_		0.212* (4.96)			0.143** (5.715)	
	ER_3_			0.063 (-2.13)			− 0.146** (-4.45)
	Con	YES	YES	YES	YES	YES	YES
	N	3930	3930	3930	3930	3930	3930
	R^2^	0.566	0.484	0.345	0.099	0.053	0.054

### 5.4 Heterogeneity analysis

This article intends to study the mechanism of environmental protection legislation on the innovation of green industry clusters from four perspectives: region, ownership, factor density, and industry. For emerging economies like China, the study of heterogeneity is particularly important. Governments of various countries can formulate specific environmental policies by studying these issues.

#### 5.4.1 Heterogeneity of regions

There are significant differences in the economic development level, resource endowment, industrial structure, and other aspects among various provinces and cities in China. If the study of actual policies cannot take into account the significant differences between regions, then this conclusion will become less important. This article takes 30 provinces in China as an example and draws on the experience of China’s economic development to divide them into three parts: East, Central, and West. The economic development level in eastern China is relatively high, with a high degree of marketization, a well-developed industrial structure, and a high level of economic development. Next is the central region with the lowest level of economic development.

Based on this, the impact of environmental restrictions in various locations on firm GTI is explored, and the findings are provided in Table 5. In the green invention patent model in the eastern region, the coefficient of ER1 was 0.053, which passed the 10% significance level test, indicating that ER1 had a positive promoting effect on green invention patents; The coefficient of ER2 was 0.374, which passed the significance level test of 5%, indicating that ER2 had a significant positive impact on green invention patents; The coefficient of ER3 was -0.263, which passed the significance level test of 5%, indicating that the impact of ER3 on green invention patents was significantly negative. In the green utility model patent model in the eastern region, the coefficient of ER1 was 0.085, which passed the significance level test of 5%; The coefficient of ER2 was 0.526, which passed the significance level test of 5%, indicating that ER2 had a significant positive impact on green utility model patents; The coefficient of ER3 was -0.412, indicating a significant negative impact of ER3 on green utility model patents.According to the regression results, environmental regulation has a considerable impact on the GTI of companies in the Eastern sample, with ER1 and ER2 both favoring the growth of green invention patents and green utility model patents. In terms of intensity, ER2 has a stronger effect than ER1, although ER3 inhibits GTI activity significantly. In the central region, only ER1 and ER3 had a significant impact on green utility model patents. The coefficient of impact of ER1 on green utility model patents was -0.245, indicating that the impact of ER1 on green utility model patents was negative at the 10% significant level. The coefficient of influence of ER3 on green utility model patents was 0.445, indicating that the impact of ER3 on green utility model patents was positive at a significant level of 5%. In the western region, ER1, ER2, and ER3 were not significantly affected by green invention patents and green utility model patents, Indicating that none of the three environmental regulatory methods had a substantial impact on GTI.

**Table 5:** Regression results of heterogeneity of regions.

		green invention patent			green utility model patent		
		ER_1_	ER_2_	ER_3_	ER_1_	ER_2_	ER_3_
East	coefficient	0.053* (2.12)	0.374** (4.33)	-0.263** (-3.02)	0.085** (3.58)	0.526** (8.23)	-0.412** (-6.12)
	_cons	-2.956* (-5.23)	-2.956** (-8.42)	-2.965** (-8.23)	-2.036** (-5.43)	-1.995* (-4.42)	-1.112** (-5.23)
	Con	YES	YES	YES	YES	YES	YES
	N	2800	2800	2800	2800	2800	2800
	R^2^	0.413	0.415	0.412	0.096	0.146	0.425
Central	coefficient	-0.156 (-1.95)	-0.098 (-0.86)	0.956 (0.77)	-0.245* (-3.85)	0.988 (-0.42)	0.445** (3.26)
	_cons	0.196** (-2.85)	-1.332** (-2.75)	-3.452** (-4.23)	0.495 (0.51)	0.436 (0.51)	-0.055 (0.85)
	Con	YES	YES	YES	YES	YES	YES
	N	660	660	660	660	660	660
	R^2^	0.413	0.362	0.485	0.456	0.238	0.196
West	coefficient	0.039 (0.62)	-0.085 (-0.95)	-0.096 (-0.87)	0.039 (0.36)	-0.053 (-3.52)	-0.142 (-1.85)
	_cons	-2.856** (-5.23)	-3.112* (-4.96)	-2.745** (-5.93)	-2.236** (-2.85)	-2.125** (-5.23)	-2.306* (-4.03)
	Con	YES	YES	YES	YES	YES	YES
	N	530	530	530	530	530	530
	R^2^	0.224	0.256	0.236	0.362	0.159	0.223

Through the comparison of these three regions, it can be found that the strict environmental rules in the eastern region help to promote innovation. Under the dual role of government supervision and market incentives, enterprises can better adapt to the production mode and strive to achieve green development. However, due to the backward industrial structure in the central and western regions, its environmental regulatory policies make it difficult to promote the growth of corporate GTI. The industrial development of the central and western regions mainly depends on the extensive resource utilization model, and with the transfer of high pollution and high energy-consuming industries to the central and western regions, enterprises need to continue to strive to get rid of the extensive development model. Therefore, enterprises in the central and western regions may face insufficient GTI investment under the implementation of environmental regulation policies, which may even lead to the migration of enterprises from the central and western regions to regions with weak environmental regulation.

By studying the impact of environmental restrictions in different regions of China on corporate GTI, it reveals the differences in economic development and environmental management among provinces in China, and points out that the eastern region has advantages in green innovation, while the central and western regions face some challenges due to industrial structure problems. This is of great significance for formulating targeted regional policies and promoting sustainable development.

#### 5.4.2 Heterogeneity of ownership

State-owned enterprises and non-state enterprises collectively constitute a substantial portion of China’s national economy. There are noteworthy distinctions between them concerning their nature, primary responsibilities, and business objectives, which represent a crucial matter that necessitates attention in the study of China’s economy.State owned enterprises are the representatives of state-owned assets and the concentrated embodiment of national will. They not only aim to meet the profits of enterprises, but also demonstrate the government’s control over all aspects of economic and social development. However, private enterprises aim to maximize economic benefits. For the purposes of this paper, the sample firms are divided into state-owned and non-state-owned firms in order to analyze the impact of environmental restrictions on the GTI heterogeneity of firms with different ownership characteristics. The regression results are shown in Table 6. In state-owned enterprises, the coefficient of influence of ER2 on green invention patents is 0.275, and it is significantly positive at the 5% level. The impact coefficient of ER2 on green utility model patents is 0.144, and it is significantly positive at the 10% level. The impact coefficient of ER3 on green utility model patents is -0.299, and it is significantly negative at the 10% level. In non-state-owned enterprises, the coefficient of influence of ER1 on green utility model patents is 0.094, and it is significantly positive at the 10% level. The impact coefficient of ER3 on green utility model patents is -0.263, and it is significantly negative at the 10% level.

**Table 6:** Regression results of heterogeneity of ownership.

		green invention patent			green utility model patent		
		ER_1_	ER_2_	ER_3_	ER_1_	ER_2_	ER_3_
state-owned enterprise	coefficient	0.056 (1.24)	0.275** (3.63)	− 0.036 (0.39)	0.036 (0.95)	0.144* (2.002)	−0.299* (0.816)
	_cons	− 2.03*** (-8.03)	−3.563** (-9.62)	−2.95*** (-8.02)	−2.035** (-4.29)	−1.612** (-6.13)	−1.422** (-6.99)
	Con	YES	YES	YES	YES	YES	YES
	N	2800	2800	2800	2800	2800	2800
	R^2^	0.392	0.549	0.594	0.057	0.041	0.062
non-state-owned enterprises	coefficient	0.023 (1.54)	0.037 (0.49)	− 0.084 (-0.66)	0.094** (2.13)	0.195 (2.03)	− 0.263* (-4.239)
	_cons	− 2.003** (-4.91)	−2.116** (-4.92)	−2.023*** (-4.32)	−2.654** (-1.556)	−1.046** (-3.38)	− 1.974* (-2.23)
	Con	YES	YES	YES	YES	YES	YES
	N	1900	1900	1900	1900	1900	1900
	R^2^	0.364	0.749	0.716	0.083	0.064	0.288

From the regression findings, it can be deduced that: (1) ER1 strongly enhances the patent level of the green utility model in a sample of non-state owned enterprises, but has no effect on green invention patents.The research results indicate that under ER1 conditions, non-state-owned companies are more inclined to adopt green technologies to compensate for the losses caused by environmental pollution. Compared with state-owned enterprises, private companies have greater flexibility in adjusting their own development strategies and have a greater promoting effect on green technology innovation. In the process of adopting green technology innovation, private enterprises often encounter green practical technologies with lower technical difficulty. (2) ER2 has a promoting effect on the technological innovation of state-owned enterprises (including green inventions and green utility models), indicating the promoting effect of ER2 on the technological innovation of state-owned enterprises. (3) The ER3 effect has had a negative impact on both types of enterprises, and green utility model products have a strong inhibitory effect on their patents. It can be shown that green utility models, regardless of the ownership of the business, are more vulnerable to external public opinion than green inventions.

#### 5.4.3 Heterogeneity of factor intensity

When facing environmental regulations, enterprises with significant differences in factor density exhibit heterogeneity in their choices of green technology innovation. For example, relying on resource intensive companies for transformation is usually better than relying on labor-intensive companies. Based on Dong and Guo’s research [47]. The article categorizes listed companies in China into technology intensive, capital intensive, labor-intensive, and resource intensive. Then, the impact of environmental regulations on the differences in green innovation capabilities of four types of enterprises was studied. The results are shown in Table 7. For technology intensive enterprises, in the regression model of green utility model patents, the coefficient of the independent variable ER1 is 0.063, which passes the 5% significance level test, indicating a positive relationship between ER1 and green utility model patents. The coefficient of ER3 is -0.185, which passes the significance level test of 5%, indicating that the impact of ER3 on green utility model patents is significantly negative.For capital intensive enterprises, the coefficient of influence of ER1 on green invention patents is 0.052, which passes the significance level test of 5%, indicating that ER1 has a significant positive impact on green invention patents. The coefficient of influence of ER2 on green invention patents is 0.56, which passes the significance level test of 1%, indicating that ER2 has a significant positive impact on green invention patents. The coefficient of influence of ER1 on green utility model patents is 0.055, which passes the 1% significance level test, indicating a positive relationship between ER1 and green utility model patents.For labor-intensive enterprises, in the regression model of green invention patents, indicating a negative relationship between ER1 and ER3 and green invention patents, and a positive relationship between ER2 and green invention patents.For resource intensive enterprises, there is no significant relationship between ER1, ER2, ER3 and green invention patents or green utility model patents.

**Table 7:** Regression results of heterogeneity of factor intensity.

		green invention patent			green utility model patent		
		ER_1_	ER_2_	ER_3_	ER_1_	ER_2_	ER_3_
technology-intensive	coefficient	0.039 (2.36)	0.085 (1.22)	-0.085 (-1.62)	0.063** (2.95)	0.223 (1.85)	-0.185** (-2.95)
	_cons	−2.036** (-4.52)	-1.859* (-5.03)	-1.665* (-3.26)	-1.856* (-3.95)	-1.956* (-3.26)	-1.455** (-4.23)
	Con	YES	YES	YES	YES	YES	YES
	N	1760	1760	1760	1760	1760	1760
	R^2^	0.385	0.485	0.322	0.096	0.085	0.062
capital-intensive	coefficient	0.052** (2.42)	0.56*** (3.96)	0.003 (0.04)	0.055** (1.95)	0.185 (1.88)	-0.085 (-2.85)
	_cons	-2.512** (-6.84)	-2.144* (-6.88)	-2.856* (-5.23)	-2.036* (-4.96)	-1.123** (-4.88)	-1.612** (-6.23)
	Con	YES	YES	YES	YES	YES	YES
	N	1500	1500	1500	1500	1500	1500
	R^2^	0.362	0.488	0.144	0.082	0.086	0.086
labor-intensive	coefficient	-0.08* (-2.12)	0.236** (3.85)	-0.596* (0.466)	-0.036 (-0.85)	0.485** (2.89)	-0.599** (-5.23)
	_cons	-3.856* (-4.023)	-3.223* (-4.96)	-3.026* (-5.23)	-1.235** (-3.84)	-1.965* (-2.88)	-1.441 (0.523)
	Con	YES	YES	YES	YES	YES	YES
	N	790	790	790	790	790	790
	R^2^	0.342	0.146	0.565	0.442	0.155	0.416
resource-intensive	coefficient	-0.163 (-1.84)	0.096 (0.22)	-0.043 (-0.84)	0.098 (0.42)	0.035 (0.43)	-0.032 (-0.54)
	_cons	-4.856* (-4.96)	-4.589* (-5.23)	-4.023* (-6.23)	-2.236 (-1.445)	-2.036 (0.88)	-1.956 (-3.26)
	Con	YES	YES	YES	YES	YES	YES
	N	400	400	400	400	400	400
	R^2^	0.286	0.326	0.395	0.563	0.196	0.463

Through empirical analysis, it was found that: (1) ER1 plays an important role in enhancing the green innovation capabilities of high-tech and capital intensive enterprises, while it has a significant restraining effect on the improvement of the green innovation capabilities of labor-intensive enterprises. One of the reasons for this is that both technology and capital intensive enterprises have higher technological innovation and adaptability. When facing mandatory environmental control policies, they can quickly determine the technological innovation priority of green industry clusters, thereby gaining technological competitive advantages. In the production process, labor-intensive enterprises have low technological innovation capabilities and environmental awareness, and their willingness to purchase green machinery products is higher than that of green manufacturing enterprises. (2) ER2 has a significant promoting effect on green innovation in capital intensive and labor-intensive enterprises. The promotion effect of ER2 on capital intensive enterprises is similar to that of ER1. In capital intensive enterprises, in order to avoid taxes, their ability to seek flexible technological innovation will encourage them to engage in green technological innovation. Compared to ER1, ER2 bears much less pressure on labor-intensive enterprises and can provide a buffer time for such enterprises to some extent, thereby promoting the development of green technology innovation.(3) Research has found that in China, especially labor-intensive enterprises, they often adopt short-term terminal governance strategies when facing public opinion supervision. Overall, the activity of ER2 is continuously increasing, while the activity of ER3 is mainly inhibited, indicating that the environmental awareness of the Chinese people needs to be strengthened. The three environmental laws and regulations have little effect on promoting the green development of resource-based enterprises.One probable explanation is that the production activities of resource-intensive enterprises rely mostly on natural resources.In addition, environmental rules had minimal impact on the GTI due to the poor innovation base and the relative difficulty of modifying the production process.

## 6 Mechanism test

This section will conduct in-depth research on the environmental regulatory processes that affect the company’s green development strategy. On this basis, this article proposes the impact of environmental legislation on the innovation performance of green industries, which is achieved through two methods: internal financing and external financing of enterprises. Therefore, this project will analyze it from two levels: endogenous (R&D) and external (government support). On this basis, Indicators such as TFP and lagging environmental regulation were selected through our analysis and analyzed their mechanisms from the perspectives of marketization and time.

### 6.1 The moderating effect of enterprise R&D investment

The model (5) developed in Section 4.3 is used to run the regression on the moderating effect of corporate R&D spending, and the results are shown in Table 8.The basic meaning of each column is consistent with the previous text. From the test results of three different types of environmental regulations, it can be seen from Table 8 that enterprise R&D investment positively regulates the relationship between ER2 and green inventions at a significance level of 5%, and positively regulates the relationship between ER2 and green utility models at a significance level of 5%.The regression results demonstrate that R&D spending considerably moderates the connection between ER2 and Green innovation patents and ER2 and Green utility model patents. These findings suggest that ER2 empowers enterprises to make their own judgements when it comes to internalising the cost of pollution. Companies have implemented GTI during this period by increasing investment in research and development, thereby improving resource usage, reducing energy consumption and reducing pollution control expenses.The possible reason is that market-oriented environmental regulations use emission permits, emission trading, and other economic tools; Establish pollutant emission prices or taxes, and encourage enterprises to adopt environmental protection behaviors. For some manufacturing enterprises, in order to reduce pollution emissions, they increase research and development investment, develop new technologies or improve existing technologies to reduce environmental impacts during the production process. Environmental regulations typically encourage companies to invest in green technology innovation to comply with these regulations and protect their market position. At a time when environmental protection and sustainable development have become key global issues, the relationship between environmental regulation (ER) and green technology innovation (GTI) in enterprises has become particularly important. R&D investment is often seen as a key factor driving technological innovation, while also having a moderating effect on the relationship between environmental regulations and corporate GTI. Therefore, the increase in R&D investment by enterprises has a positive regulatory effect on invention patents. In other words, increased R&D investment can not only directly improve the quantity or quality of green technology innovation, but also enhance the promoting effect of environmental regulations on GTI.

**Table 8:** Regression results of the moderating effect of corporate R&D investment.

Variables	Green invention patent				Greenutility modelpatent			
	(1)	(2)	(3)	(4)	(5)	(6)	(7)	(8)
ER1	0.053 (1.85)			0.059** (3.125)	0.036** (3.95)			0.092** (4.45)
ER2		0.195** (2.42)		0.296** (3.42)		0.095*** (2.43)		0.289** (5.96)
ER3			−0.169** (-3.02)	-0.166* (-2.54)			-0.145** (-2.36)	-0.096** (4.462)
RD	0.028* (5.85)	0.043* (1.55)	0.046** (6.85)	0.004 (1.44)	0.023** (3.635)	−0.09** (-1.58)	0.003* (2.95)	0.003 (1.96)
ER1 × RD	0.003 (0.89)			−0.004 (-1.85)	−0.003** (0.856)			−0.002 (0.196)
ER2 × RD		0.008** (2.55)		0.008 (3.69)		0.046** (4.85)		-0.185 (4.039)
ER3 × RD			0.005 (1.45)	0.013* (3.565)			−0.024** (-3.695)	−0.023 (-0.88)
_cons	-2.036* (-8.85)	-2.463** (-8.45)	-4.03* (-8.96)	-2.455* (-8.13)	-1.956** (0.85)	-1.956** (-5.23)	0.584* (-3.023)	-1.845** (-6.23)
Con	YES	YES	YES	YES	YES	YES	YES	YES
N	3930	3930	3930	3930	3930	3930	3930	3930
R2	0.152	0.499	0.526	0.152	0.023	0.055	0.167	0.362

Karmaker [48] used a globally robust model with significant statistical capabilities to study the causal relationship between environmental taxes and environmental related technological innovation. This model uses panel cointegration analysis considering cross-sectional dependence to conduct a quantitative study on 42 high-income and middle-income countries from 1995 to 2018, and finds that the implementation of environmental taxes has stimulated technological innovation. Levying environmental taxes can help accelerate the reduction of carbon emissions and promote the development of green innovative technologies in high-income and middle-income countries. Meanwhile, Shang et al [31], who share the same view, conducted an empirical test on the impact of environmental tax collection on corporate technological innovation. Research has found that levying environmental taxes can stimulate the innovation drive of enterprises and significantly increase the input and output of technological innovation. By taxing pollution emissions from enterprises, they will be forced to think about how to reduce environmental pollution in the production process, which can guide them to invest more in green innovation. This will encourage companies to invest more resources in green innovation. Not only can it reduce taxes and fees generated by pollution emissions, but it can also promote green technology innovation in enterprises.

At the same time,the relationship between R&D investment on environmental regulations and green innovation patents can be adjusted in three ways. (1) Increase government supervision: strengthen the supervision of enterprises to comply with environmental regulations, ensure that enterprisesare in accordance with the provisions of R&D investment, and truly achieve green innovation. This can be achieved by increasing law enforcement and increasing penalties. (2) Provide incentive mechanism: encourage enterprises to increase R&D investment, through incentives and incentives to promote enterprises to actively carry out green innovation. For example, the government can set up a green innovation fund to provide financial support for enterprises that comply with environmental regulations and carry out green innovation. Third, strengthen information disclosure and public supervision: strengthen the information disclosure of corporate R&D investment and green innovation, so that the public can understand the behavior of enterprises, and promote enterprises to better fulfil their environmental responsibilities through public opinion supervision.

These results suggest that firms that actively promote substantial green change or innovation by expanding their R&D investments may be better positioned to meet compliance requirements under government-mandated environmental regulations.

### 6.2 The moderating effect of government support

The regression on the moderating effect of government support was run using the model (6) developed in Section 4.3, and the results are reported in Table 9.On this basis, this study found that under the interaction between environmental regulation and environmental regulation, the interaction between environmental regulation and environmental regulation is both positive, but not significant. The results of this study indicate that government support may have a partial impact on the correlation between environmental regulations and corporate green invention patents, although the magnitude of its positive regulatory effect is still unclear.Regarding the regression results, government support exhibits a significant negative moderating effect on the relationship between ER1 and firms’ green utility model patents, ER3 and enterprise green utility model patents. Simultaneously, government support significantly positively moderates the relationship between ER2 and enterprise green utility model patents.The empirical findings above suggest that, when accompanied by government support, environmental constraints might encourage firms to invest in green invention patents.For Chinese enterprises, government subsidies or financial support for their technological innovation generally play a significant role, especially for companies that can effectively improve green environmental protection technologies. This study will help explain the positive regulation of environmental regulations by the government, as well as the correlation between the feedback effect of manufacturers on environmental concept patents. On the other hand, green utility model patents indicate that companies have made a short-term technological investment in order to passively comply with regulations, and their nature is closer to the "compliance cost". Therefore, when the government subsidizes "green innovation" in enterprises, compliance pressure on businesses is alleviated, granting them more buffer space to address the pressures imposed by environmental regulations. Through this approach, the research and development efforts of green utility models can be reduced, while also increasing the development scale of green invention patents.

**Table 9:** Regression results of the moderating effect of government support.

Variables	Green invention patent				Greenutility modelpatent			
	(1)	(2)	(3)	(4)	(5)	(6)	(7)	(8)
ER1	0.045 (2.03)			0.046** (2.95)	0.052* (3.43)			0.055** (3.88)
ER2		0.196** (3.85)		0.345** (4.452)		0.123** (2.96)		0.389** (4.45)
ER3			0.362** (-2.45)	-0.123** (-2.58)			0.785** (-4.75)	-0.136* (1.152)
Sub	0.028** (1.452)	0.032 (1.84)	0.023** (2.95)	0.056 (0.56)	0.212* (2.98)	−0.005 (-0.85)	0.063* (2.63)	0.062 (0.88)
ER1 × Sub	0.006 (0.51)			−0.004 (-0.96)	−0.034** (-0.95)			−0.03 (-0.95)
ER2 × Sub		0.056 (1.02)		0.058 (1.95)		0.0854** (2.85)		0.073 (1.93)
ER3 × Sub			0.006 (1.84)	0.023** (2.45)			−0.004* (-5.03)	0.005 (-0.81)
_cons	-1.856** (-8.03)	-3.565** (-9.11)	-3.845** (-9.23)	0.995* (-8.45)	-1.013** (-4.85)	-2.036** (0.84)	-0.885** (-3.41)	-1.896* (-5.42)
Con	YES	YES	YES	YES	YES	YES	YES	YES
N	3930	3930	3930	3930	3930	3930	3930	3930
R2	0.346	0.412	0.526	0.845	0.077	0.123	0.086	0.002

### 6.3 Transformation mechanism of innovation achievements

According to Porter’s theoretical hypothesis, environmental regulation has a certain impact on a company’s technological innovation, with the goal of improving the company’s production efficiency. Taking into account all the factors discussed above,it is natural to reach the conclusion that whether production efficiency can ultimately be improved is a critical criterion for enterprises seeking green technological innovation.Therefore, the purpose of this article is to explore the underlying theoretical logic of whether environmental regulations can promote the improvement of production efficiency and incentivize enterprises to implement green technology innovations from the perspectives of environmental regulation, production efficiency enhancement, and green technology innovation. Specifically, the impact of environmental restrictions on overall green factor productivity is investigated using empirical evidence. The first step is to construct the green productivity index and refer back to the methods of Wang and Zhang [49] and Kuang and Peng [50]. Among them, labor input (measured by the average number of employees in each province), capital investment (measured by net fixed assets), energy input (measured by converting regional total energy consumption into standard coal), and three output indices (measured by value added) and employment (measured by the actual number of employees in each province) are included (measured by the annual number of employees). Using the data envelopment analysis method, the total green production efficiency of each province was calculated. Finally, regression analysis was conducted on green TFP using environmental regulation methods. Table 10 showed the relationship between environmental regulations and green total factor productivity. Empirical analysis shows that ER2 has a significant driving effect on the improvement of total factor productivity in enterprises. Although both ER1 and ER3 have a significant positive relationship with green production efficiency, this result indicates that environmental regulation can improve green production efficiency by enhancing the productivity of enterprises. Another interpretation of this result is that while environmental regulations are conducive to an increase in green patents, a lack of green innovation output transformation may be an important underlying reason for inhibiting the implementation of green technology innovation by businesses, as reflected in ER1 and ER3.

**Table 10:** The relationship between environmental regulations and green total factor productivity.

Variables	(1)	(2)	(3)	(4)
ER1	0.026 (2.85)			0.069 (2.34)
ER2		0.488** (4.42)		0.1485** (5.445)
ER3			0.052 (0.63)	0.005 (0.96)
_cons	1.163** (19.24)	0.955** (14.13)	1.195** (18.125)	1.885** (15.42)
Con	YES	YES	YES	YES
N	360	360	360	360
R2	0.085	0.196	0.068	0.326

### 6.4 The temporal effects of environmental regulatory policies

When evaluating the exogenous effects of environmental regulation, the first thing to consider is its impact on policies, while also examining the speed of its impact. This section will explore the short-term and long-term effects of different environmental control measures on the company’s green technology innovation. The article takes the period of China’s Five Year Plan as an example to analyze five lagged variables. Table 11 shows the results of the regression analysis. Observe through regression coefficients.It can be determined that the influence of environmental regulations on the GTI of businesses has been decreasing over timeSpecifically, there was no statistically significant difference in the regression analysis of ER1 lag values, and in the second lag stage, there was a negative effect related to the forced ER1 trait. After adopting ER1, the company tends to quickly adjust its production process to avoid punishment, but this process will gradually weaken. The ER2 in the first and third stages has a significant promoting effect on the enthusiasm of companies for green investment, but in the fifth stage, it has a negative promoting effect on corporate green investment. Therefore, the role of ER2 in the company’s green technology innovation is likely to be only a short-term effect. ER3 has a negative effect on enterprise GTI, but under various time delayed variables, this effect gradually decreases over time. Therefore, It is speculated that the role of ER3 in promoting green technology innovation in enterprises still needs further long-term research, and the driving effect of ER3 on green technology innovation in enterprises will continue for a longer time. Overall, China’s environmental regulatory policies have a more short-term impact on the green development of enterprises. ER1 has the most rapid impact on enterprise GTI,while ER3 may play a more favorable role over a longer period of time.

**Table 11:** Results of the lag period of heterogeneous environmental regulations.

		Green invention patent			Green utility model patent		
		ER_1_	ER_2_	ER_1_	ER_2_	ER_1_	ER_2_
Lag one phase	coefficient	0.052 (2.96)	0.295** (5.023)	0.388* (1.526)	0.085 (1.462)	0.196** (2.23)	−0.062* (3.845)
	_cons	− 2.856** (-7.45)	−3.845** (-8.03)	−1.856** (-5.01)	−1.485** (-4.42)	−1.016** (-3.545)	−1.101* (-2.884)
	Con	YES	YES	YES	YES	YES	YES
	N	3562	3562	3562	3562	3562	3562
	R^2^	0.452	0.241	0.496	0.062	0.086	0.126
Lag two phases	coefficient	− 0.001 (-0.08)	0.085 (1.45)	− 0.073 (0.82)	-0.686 (-0.36)	0.038 (1.92)	− 0.008 (0.03)
	_cons	− 3.632** (-563)	−1.986** (-6.03)	−2.126* (-6.856)	− 0.941 (0.496)	0.699* (0.535)	− 0.984* (-2.46)
	Con	YES	YES	YES	YES	YES	YES
	N	2700	2700	2700	2700	2700	2700
	R^2^	0.136	0.185	0.313	0.863	0.056	0.056
Lag three phases	coefficient	− 0.051 (-0.82)	0.343** (2.88)	0.096 (2.06)	−0.295* (-5.45)	0.362* (2.445)	0.036 (0.52)
	_cons	0.945** (-4.36)	0.684** (-5.441)	-3.856** (0.859)	− 0.023 (0.155)	0.1512 (-0.62)	0.685 (-0.31)
	Con	YES	YES	YES	YES	YES	YES
	N	2976	2976	2976	2976	2976	2976
	R^2^	0.416	0.184	0.046	0.095	0.052	0.049
Lag four phases	coefficient	0.035 (0.25)	0.185 (1.96)	− 0.032 (0.195)	− 0.164 (-0.46)	0.136* (3.82)	− 0.036 (-0.81)
	_cons	− 1.846** (-3.41)	−0.984** (-3.42)	−1.511* (-5.03)	0.856 (1.95)	0.912 (3.82)	0.895 (2.35)
	Con	YES	YES	YES	YES	YES	YES
	N	2680	2680	2680	2680	2680	2680
	R^2^	0.071	0.043	0.068	0.046	0.045	0.053
Lag five phases	coefficient	− 0.063** (-3.12)	-0.084 (-1.54)	− 0.094 (0.946)	0.385** (-6.13)	− 0.213 (-1.845)	−0.296** (0.385)
	_cons	-0.956 (0.412)	0.386 (-0.89)	-0.302 (-0.46)	3.856** (6.32)	4.012** (5.63)	4.416** (6.122)
	Con	YES	YES	YES	YES	YES	YES
	N	2100	2100	2100	2100	2100	2100
	R^2^	0.036	0.063	0.023	0.645	0.158	0.462

## 7 Conclusion

China is in the early stages of transforming into a high-quality development. By formulating environmental regulations to achieve environmental protection and economic development, it is conducive to the technological upgrading of enterprises and the industrial transformation of our country. On the one hand, in-depth exploration of the mechanism of green innovation from both theoretical and empirical perspectives is of great theoretical and practical significance for the government to formulate green innovation policies, promote green innovation, promote green innovation, and promote the development of green innovation. On the other hand, this paper is a study and analysis of China, a major developing country, and it is important for other developing countries to be enlightened and referred to in the process of implementing environmental regulations.Our study empirically examines the impact of environmental pollution regulation methods on the GTI of Chinese listed companies and analyzes their mechanisms in terms of internal and external integration.Using standard regression and mediation effect models, explore the impact of environmental regulation on corporate green innovation behavior. he robustness test was performed by substituting variables and adding control variables, and the results are in general agreement with the regression results. Finally, heterogeneity analysis and institutional tests are performed. The main conclusions of this study are as follows:

1. When the three types of environmental regulation policies work at the same time, the positive impact of ER2 on corporate GTI is greater than that of ER1 and ER3, but all of them promote the GTI of enterprises to a certain extent. The three environmental regulation methods are introduced into the regression model at the same time. Compared with the introduction of a single regulatory method, it is found that the three environmental regulations work better together.
2. In this study, the heterogeneity analysis was conducted. In the eastern region, environmental control policies have a significant promoting effect on corporate green innovation. But it is difficult to cultivate enterprises in the central and western regions.ER1 has a positive effect on the low technical difficulty R&D of GTI in non-state-owned enterprises, ER2 has a positive promoting effect on the research of green technology innovation and the development of state-owned enterprises, while ER3 will have a negative impact on the research and development of green technology innovation. ER1 has hindered the green innovation development of technology intensive and capital intensive enterprises;ER2 promotes the GTI for capital-intensive and labor-intensive enterprises; ER3 plays an inhibitory role in various factor-intensive activities.
3. R&D investment plays a mediating role in ER2 and technological innovation, and there is a positive correlation between the two. There is a significant negative correlation between ER3 and ER1. Government support policies have a positive regulatory effect on the correlation between environmental regulations and company green invention patents, but this conclusion needs further exploration. The government support policies have a comprehensive negative regulatory effect on environmental regulations and corporate green utility model patents. Environmental regulations can drive green innovation by increasing a company’s productivity.
4. The impact of China’s environmental regulations on green technology innovation is mainly short-term, while the impact of environmental regulations on green technology innovation shows a decreasing trend. ER1 has the fastest impact on corporate GTI, while ER3 may play a beneficial role in a longer period of time.

## 8 Policy recommendations and future resarch directions

### 8.1 Policy recommendations

Our research has specific guiding significance in various aspects, as shown below;

For decision makers, decision makers can further promote corporate GTI by strengthening the formulation and implementation of environmental regulatory policies. At the same time, decision makers can also formulate targeted environmental regulatory policies according to the heterogeneity of region, ownership structure and factor concentration, so as to better promote the development of GTI.

For enterprises, enterprises can add green environmental protection technology to the new strategy, increase R&D investment, and carry out economic transformation. At the same time, policy adjustments can be made according to heterogeneity, and long-term development strategies can be formulated to better adapt to the changes brought about by environmental regulatory policies.

For stakeholders, environmental protection organizations, consumers, etc., can supervise the environmental situation of enterprises, so as to improve the implementation of corporate environmental policies. This not only promotes the green development of enterprises, but also improves the sustainable development and environmental quality of the whole society.

### 8.2 Future resarch directions

Future research can be conducted in the following directions, as shown below;

1. Promoting the application of green innovation technology: Encourage and support enterprises to carry out green innovation technology and promote sustainable development. By employing measures such as government financial support and tax incentives, intensifying support for the development and application of green innovation technologies can, in turn, stimulate businesses to embrace technologies that are not only ecologically friendly but also highly efficient.
2. Economic incentives: Establish a sound economic incentive mechanism, such as environmental taxes, emissions trading, etc., to guide enterprises to reduce pollution and save resources, so that enterprises are economically aware of the impact of the environment, so as to take environmental protection measures.
3. Guiding public participation: strengthen the transparency of corporate information and encourage public participation in environmental decision-making and supervision. By instituting a comprehensive and transparent environmental data platform, the general populace gains access to insights into corporate environmental performance, along with offering channels and commensurate incentives for reporting, thereby fostering augment societal oversight and corporate accountability.
4. In line with international standards: Actively participate in international environmental regulation activities, share and learn experience and technology with other countries and regions, and jointly respond to global environmental challenges. At the same time, it should be in line with international standards, improve the standardization level of domestic environmental regulation, and promote the competitiveness of enterprises in the global market.

The research findings of this article will provide decision-making basis for the improvement of China’s environmental policies and the sustainable development of Chinese enterprises. This article is based on empirical research on China as a developing country and provides some useful suggestions for China’s environmental regulation practices. Since the reform and opening up, China has shifted from a planned economy to a market economy, and the market mechanism has played a good regulatory role in the Chinese economy. For other developing countries, when implementing market-based environmental regulation systems, they should adjust their enforcement concepts in a timely manner based on the actual operation of their own market economy, in order to avoid the abuse of market control tools due to imperfect market mechanisms and poor policy effects.

## Supporting information

S1 File (7Z)

S2 File (ZIP)
